# Increasing Malaria Parasite Clearance Time after Chloroquine Therapy, South Korea, 2000–2016 

**DOI:** 10.3201/eid2608.190687

**Published:** 2020-08

**Authors:** Seong Yeon Park, Yoon Soo Park, Yoonseon Park, Yee Gyung Kwak, Je Eun Song, Kkot Sil Lee, Shin-Hyeong Cho, Sang-Eun Lee, Hyun-Il Shin, Joon-Sup Yeom

**Affiliations:** Dongguk University Ilsan Hospital, Gyeonggi-do, South Korea (S.Y. Park);; National Health Insurance Service Ilsan Hospital, Gyeonggi-do (Y.S. Park, Y. Park);; Inje University Ilsan Paik Hospital, Gyeonggi-do (Y.G. Kwak, J.E. Song);; Myongji Hospital, Gyeonggi-do (K.S. Lee);; Korea Centers for Disease Control and Prevention, Chungcheongbuk-do, South Korea (S.-H. Cho, S.-E. Lee, H.-I. Shin);; Yonsei University College of Medicine, Seodaemun-gu, Seoul, South Korea (J.-S. Yeom)

**Keywords:** Chloroquine, fever clearance time, hydroxychloroquine, malaria, parasite clearance time, *Plasmodium vivax*, primaquine, South Korea, retrospective studies, parasites

## Abstract

We reviewed the clinical efficacy of chloroquine for *Plasmodium vivax* malaria, the changing trend of parasite clearance time, and fever clearance time during 2000–2016 in South Korea. Median parasite clearance time and fever clearance time increased significantly over the study period. Chloroquine was mostly underdosed when used to treat *P. vivax* malaria.

*Plasmodium vivax* is the only form of malaria indigenous to South Korea (*1*); combination chloroquine and primaquine therapy has been the mainstay of vivax malaria treatment. After *P. vivax* reemerged after being eliminated in South Korea in the late 1970s, it mainly occurred in soldiers or veterans stationed in the demilitarized zone between North Korea and South Korea. Because of this focus, mass chloroquine chemoprophylaxis has been administered in place for soldiers working in high-risk areas since 1997 (*2*,*3*). However, there is concern that mass chemoprophylaxis might lead to reduced chloroquine susceptibility (*2*). In addition, soldiers were often given lower doses than that recommended by the World Health Organization (WHO); Commons et al. reported that underdosing of chloroquine was associated with recurrence and increased parasite clearance time (PCT) (*4*). We studied the trends in South Korea during 2000–2016 for treatment efficacy on the basis of PCT and fever clearance time (FCT). 

## The Study 

We conducted this study in 4 hospitals in Goyang, Gyeonggi Province, and 1 hospital in Seoul. The study population consisted of patients ˃18 years of age in whom *P. vivax* malaria had been diagnosed during 2000–2016 and who had been prescribed chloroquine alone or in combination with primaquine. We determined PCT, defined as the time in hours from chloroquine administration until the first blood smear negative for parasites after which the patient’s follow-up smears were also negative, from malaria smears taken daily in routine treatment of patients. To determine FCT, defined as the time from chloroquine administration until the patient’s body temperature decreased to ˂37.5°C for 48 consecutive hours (*5*), we checked for fever every 4–6 hours or whenever the patient felt febrile. 

This study was approved by the local institutional review board. Statistical analyses were performed with SPSS Statistics 20 (IBM, https://www.ibm.com). For PCT and FCT, we reported median and interquartile ranges (IQR) because Shapiro-Wilk test results showed that outcome measures did not follow a normal distribution. We compared trend analyses for PCT and FCT by enrollment periods using a linear-by-linear association. 

We analyzed included data from a total of 1,199 malaria cases over the 17-year study period. The mean (+SD) age of patients was 41.7 +14.2 years. None of the patients died of malaria during the study period; 44 relapsed. We divided the years of the study period into 3 ranges. During 2000–2005, 404 malaria cases occurred; that number increased to 566 during 2006–2010, then decreased to 229 during 2011–2016. (Table 1). 

**Table 1:** Demographic and clinical data for patients with *Plasmodium vivax* malaria, by study period, South Korea, 2000–2016*

Variable	Years			p value
	2000–2005, n = 404	2006–2010, n = 566	2011–2016, n = 229	
Age, y, mean (±SD)	40.5 ±14.3	42.5 ±14.0	41.6 ±14.6	0.06
Sex				
M	279 (69.1)	410 (72.4)	179 (78.2)	0.045
F	125 (30.9)	156 (27.6)	50 (21.8)	
Weight, kg, mean (±SD)	66.3 ± 0.57	68.4 ± 0.53	70.5 ± 0.88	<0.001
Smear follow-up	131 (32.4)	168 (29.7)	82 (35.8)	0.23
Median PCT (IQR), h	63.0 (49.0–82.0)	66.0 (56.3–78.0)	75.0 (52.0–96.5)	<0.001
PCT >48 h	100/131 (76.3)	145/168 (86.3)	71/82 (86.6)	0.03
PCT >72 h	40/131 (30.5)	61/168 (36.3)	43/82 (52.4)	0.005
Fever	347 (85.9)	521 (92.0)	206 (90.0)	0.04
Median FCT (IQR), h	28.0 (14.0–42.0)	43.0 (29.0–59.0)	48.0 (28.0–66.0)	<0.001
FCT >48 h	49/279 (17.6)	198/477 (41.5)	87/181 (48.1)	<0.001
FCT >72 h	16/279 (5.7)	54/477 (11.3)	29/181 (16.0)	0.001
CQ, mg/kg, mean (±SD)	23.5 ±5.0	22.9 ±4.4	22.1 ±4.8	0.001
CQ <25 mg/kg	278 (68.8)	383 (67.7)	177 (77.3)	0.02
Primaquine, mg/kg, mean (±SD)	0.23 ± 0.06	0.24 ± 0.06	0.29 ± 0.12	<0.001
Primaquine <0.25	265/373 (71.0)	343/543 (63.2)	114/221 (51.6)	<0.001
Relapse	9/394 (2.3)	20/541 (3.7)	10/220 (4.5)	0.20

*Values are no. (%) patients/no. treated except as indicated. CQ, chloroquine; FCT, fever clearance time; IQR, interquartile range; PCT, parasite clearance time.

Median PCT increased from 63.0 hours during 2000–2005 to 75.0 hours during 2011–2016 (p<0.001). The proportion of patients whose PCT was ˃48 hours significantly increased over the 17 years from 76.3% during 2000–2005 to 86.6% during 2011–2016 (p = 0.03). In addition, the proportion of PCT at ˃72 hours also significantly increased from 30.5% during 2000–2005 to 52.4% during 2011–2016 (p = 0.005). 

We found fever in 1,074 (89.6%) of 1,199 patients at the time *P. vivax* malaria was diagnosed. Among those patients, median FCT increased from 28.0 hours during 2000–2005 to 48.0 hours during 2011–2016 (p<0.001). The proportion of patients whose FCT was ˃48 hours increased from 17.6% during 2000–2005 to 48.1% during 2011–2016 (p<0.001). The proportion of patients whose FCT was ˃72 hours also increased significantly from 5.7% during 2000–2005 to 16.0% during 2011–2016 (p = 0.001). 

All of the study patients were treated with either hydroxychloroquine or chloroquine phosphate. The mean total dose of chloroquine base administered was 23.0 mg/kg body weight in 838 patients (69.9%), which is less than the WHO-recommended target dose of 25 mg/kg. The trend of the mean of the total dose of chloroquine base decreased from 23.5 mg/kg during 2000–2005 to 22.1 mg/kg during 2011–2016 (p<0.001). In addition, the proportion of patients receiving less than the WHO-recommended dose increased from 68.8% during 2000–2005 to 77.3% during 2011–2016 (p = 0.02).

Comparing patients with mean total chloroquine doses of >25 mg/kg or <25 mg/kg, we found that men were more likely than women to be in the <25 mg/kg group (84.2% vs. 44.9%; p<0.001) (Table 2). Median FCT was longer in the <25 mg/kg group (65.0 h vs. 62.5 h; p = 0.02), and patients whose fever lasted ˃72 hours were more common in the <25 mg/kg group (12.1% vs. 7.8%; p = 0.03). Relapse was also more common in this group (4.7% vs. 1.4%; p = 0.003). 

**Table 2:** Comparison of clinical features for patients with *Plasmodium vivax* malaria, according to the dosage of chloroquine, South Korea*

Variable	CQ ≥25 mg/kg, n = 361	CQ <25 mg/kg, n = 838	p value
Age, y, mean (±SD)	42.6 ± 16.2	41.2 ± 13.2	0.13
Sex			
M	162 (44.9)	706 (84.2)	<0.001
F	199 (55.1)	132 (15.8)	
Positive for underlying disease	66 (18.3)	144 (17.2)	0.35
Primaquine administration	350 (97.0)	831 (99.2)	0.006
Primaquine, mg/kg, mean (±SD)	0.27 (0.25–0.30)	0.21 (0.19–0.23)	<0.001
Smear follow-up	122 (33.8)	259 (30.9)	0.18
Median PCT (IQR), h	62.5 (51.8–81.8)	65.0 (52.0–81.0)	0.19
PCT >48 h	98/122 (80.3)	215/259 (83.0)	0.31
PCT >72 h	37/122 (30.3)	91/259 (35.1)	0.21
Fever	322 (89.2)	752 (89.7)	0.43
Median FCT (IQR), h	37.0 (23.0–54.0)	38.0 (24.0–48.0)	0.02
FCT >48 h	98/295 (33.2)	238/647 (36.8)	0.16
FCT >72 h	23/295 (7.8)	78/647 (12.1)	0.03
Relapse	5 (1.4)	39 (4.7)	0.003

*Values are no. (%) patients/no. treated except as indicated. CQ, chloroquine; FCT, fever clearance time; IQR, interquartile range; PCT, parasite clearance time.

## Conclusions

In this study, we made 2 important findings. First, the trend of increased PCT and FCT in South Korea might be ascribed to decreased *P. vivax* susceptibility to chloroquine that could be seen before the emergence of WHO-recognized drug resistance. Mass chemoprophylaxis of military personnel with hydroxychloroquine started in 1997 in South Korea (*3*,*6*). However, mass chemoprophylaxis with poor compliance to recommended dosing among the patients increased the possibility of chloroquine-resistant strains of *P. vivax* emerging*.* This finding may explain the failure of the prophylactic hydroxychloroquine treatment to widely protect South Korean army soldiers from *P. vivax* malaria by 2000, chloroquine-resistant cases occurring by the early to mid-2000s (*7*), and the increase in PCT after chloroquine treatment since the late 2000s (*2*). 

Second, in areas with chloroquine-sensitive *P. vivax,* WHO recommends oral chloroquine at a total dose of 25 mg base/kg. Lower total doses are not recommended because they encourage the emergence of resistance (*8*). However, in the current study, ≈70% of patients received a suboptimal dose (<25 mg/kg) of chloroquine, partly because the total dose of chloroquine administered in South Korea was usually fixed at 1,500 mg base, as recommended by the US Centers for Disease Control and Prevention (*9*), rather than a weight-based dose. In our study, the average body weight of patients significantly increased, from 66 kg during 2000–2005 to 71 kg during 2011–2016 (p<0.001), but the recommended total fixed dose of chloroquine did not change. On the basis of the WHO recommendation of 25 mg/kg base, a total dose of chloroquine of 1,500 mg base would have been inadequate for patients whose body weight was ˃60 kg. In our study, we also found that relapse was more common in the <25 mg/kg chloroquine dose group (4.7% vs 1.4%; p = 0.003). 

This study has several limitations. First, parasite clearance was identified in only one third of study patients, but malaria smears were taken once daily, so clearance of parasites could have occurred at any hour in the interval between slides being taken, which could have affected the calculation of PCT. Second, temperature measurements might have been affected by the use of antipyretics and patients were not checked for fever hourly during hospitalization, either of which could have affected the calculation of FCT. Third, due to the retrospective nature of the study, we could not obtain other information that might affect estimates of PCT, such as patient vomiting, which indicates that the medication may not have been absorbed normally, or FCT, such as duration of fever before malaria diagnosis. Prospective studies are needed to more accurately assess whether PCT or FCT has increased. 

In summary, increased PCT and FCT might be ascribed to reduced parasite susceptibility to chloroquine that could be seen before emergence of drug resistance in South Korea. Therefore, a proactive and continuous surveillance system for PCT and FCT is needed. In addition, chloroquine is mostly underdosed in the treatment of *P. vivax* malaria in South Korea; thus, clinicians should adhere to weight-based treatment guidelines. 
